# 

**Published:** 1890-06

**Authors:** 


### CONTENTS.

### *EDITORIALS.*

| | PAGE |
|---|---|
| The Artificial Feeding of Infants, | 241 |
| Surgical Treatment of Uterine Troubles, | 243 |
| Blunders, | 245 |
| The Ward's Island "Difficulty" in New York, | 246 |
| Abuses of Quinine, | 247 |

### *MISCELLANEOUS.*

| | |
|---|---|
| A Reply to Dr. Allen's Protest. P. P. Wells, M. D., | 247 |
| Mercury, | 250 |
| Dr. T. F. Allen's "High Potency Allopathy." B. Fincke, M. D., | 251 |
| A Reply to Dr. T. F. Allen. Charles B. Gilbert, M. D., | 255 |
| "High Potency Allœpathy." Charles B. Gilbert, M. D., | 256 |
| Natrum Muriaticum. A. McNeil, M. D., | 256 |
| Hydroa, or Fever Blisters. G. M. Pease, M. D., | 263 |
| Watch the Effect. Edward Cranch, M. D., | 263 |
| An Awful Blunder. Dr. G. W. Emery, | 267 |
| The Ward's Island "Difficulty" in New York. Some Unpublished Correspondence, | 272 |
| Some Remarks on Rumex Crispus. C. Carlton Smith, M. D., | 275 |
| British Medicinal Plants. Alfred Heath, M. D., F. L. S., | 279 |
| A Case of Sporadic Cholera. L. Hoopes, M. D., | 282 |
| Clinical Cases. Ella M. Tuttle, M. D., | 283 |
| Sanicula—A Caution. D. W. Clausen, M. D., | 284 |
| A German Homœopathic Congress. Dr. Alexander Von Villers, | 285 |
| Help Wanted. Edward T. Balch, M. D., | 286 |
| La Grippe. W. A. Yingling, M. D., | 286 |
| Winter Cough. Frederic Preston, M. D., | 286 |

### *BOOK NOTICES.*

| | |
|---|---|
| The Memphis Journal of the Medical Sciences, | 287 |
| Homœopathic Therapeutics. Samuel Lilienthal, M. D., | 287 |
| The Pulte Quarterly, | 288 |
| The Dietetic Gazette, | 288 |
| The National Magazine, | 288 |

### *NOTES AND NOTICES.*

| | |
|---|---|
| Dr. Wm. T. Belfield, | 288 |

